# Post-marketing safety surveillance conducted in Korea (2008–2013) following the introduction of the rotavirus vaccine, RIX4414 (Rotarix™)

**DOI:** 10.1080/21645515.2016.1189046

**Published:** 2016-08-05

**Authors:** Son Moon Shin, Chun Soo Kim, Naveen Karkada, Aixue Liu, Girish Jayadeva, Htay Htay Han

**Affiliations:** a Dankook University College of Medicine, Seoul, Korea; b Keimyung University School of Medicine, Daegu, Korea; c GSK Pharmaceuticals, Bangalore, India; d Merck, North Wales, PA, USA; e GSK Vaccines, King of Prussia, PA, USA

**Keywords:** gastroenteritis, Korea, post-marketing surveillance, rotavirus, safety

## Abstract

**Purpose**: According to regulations from the Ministry of Food and Drug Safety in Korea, additional safety information on the use of *Rotarix™* vaccine (RIX4414; GSK, Belgium) in ≥3000 evaluable Korean infants was required following vaccine registration. In order to comply with these regulations, we conducted a 6-year open, non-comparative, multicenter post-marketing surveillance (NCT00750893). **Methods**: During this time, the original lyophilized vaccine formulation of RIX4414 was replaced by a liquid formulation. Healthy infants aged ≥6 weeks were enrolled and given 2 doses of the RIX4414 vaccine, separated by an interval of ≥4 weeks. The overall incidence of adverse events (AEs) (expected and unexpected) was then assessed for up to 30 days along with the incidence of serious adverse events (SAEs). Adverse drug reactions (ADRs: any AE whose causality to the drug could not be ruled out) were identified. **Results**: A total of 3040 children (mean age: 9.55 weeks) were analyzed. One or more expected AE was experienced by 30.5% infants and 8.6% had an ADR. The most commonly seen expected AE was irritability (14.0%). One or more unexpected AE was seen in 32.5% infants and 3.1% experienced an ADR. The most commonly seen unexpected AE was upper respiratory tract infection (8.7%). Of 34 SAEs recorded in 24 subjects, none were related to vaccination. **Conclusions**: We conclude that this 6-year surveillance showed both formulations of RIX4414 to have acceptable safety profiles when administered to Korean infants according to local prescribing recommendations and current clinical practice.

## Introduction

Rotavirus (RV) is the leading global cause of acute gastroenteritis (GE) in children <5 y of age,^1^ and accounts for 40% of all severe cases of infant diarrhea.^2^ In developing countries, RV causes around 360,000–600,000 child deaths each year^3,4^ and in more developed countries, 1 out of every 67 children <5 years of age will be hospitalized for GE sometime in their life.^5^

In Korea, RV is one of the most commonly seen pathogens in patients with acute GE.^6^ Detection rates for RV which occur specifically during the winter and spring seasons steadily increased from 2005−2007.^7,8^ The annual incidence of RV between 2002 and 2004 was approximately 56.9 cases per 1000 children.^9^

Two vaccines against RV, *RotaTeq*™ (Merck and Co, Inc., United States) and *Rotarix*™ (RIX4414, GSK, Belgium), were launched in South Korea in September 2007 and July 2008, respectively. The lyophilized formulation of RIX4414 was initially registered in Korea, but was subsequently replaced by the liquid formulation, comprising an oral suspension in a pre-filled syringe in December 2011.^10^ Trials conducted in Korea, Latin America, Africa and Europe showed that *Rotateq*™ (Merck and Co, Inc., United States) and *Rotarix*™ (RIX4414, GSK, Belgium) are both well-tolerated, immunogenic and efficacious.^11-15^ Further, these vaccines have been shown to be effective and safe, through the Korean National Immunization Program^16^ and the rates of vaccination in Korea have increased annually from 7% in 2007, to 35% in 2008 and 50% in 2009 (data based on registered vaccine sales).^17^

In accordance with regulations from the Ministry of Food and Drug Safety (MFDS) in Korea, additional safety information on the use of RIX4414 in at least 3000 evaluable Korean infants, was required following vaccine registration. This study was therefore undertaken to comply with the regulatory requirements.

## Results

### Study population

A total of 3112 infants were screened from 2008 to 2013 for participation in the study, of whom 3111 were included in the total vaccinated cohort (TVC). One infant, although allocated a subject number, was not given the vaccine. Of 3111 infants, 3040 (Dose 1 = 2461; Dose 2 = 2499) received the study vaccine as per the indicated age specified in the label and 71 (Dose = 51; Dose 2 = 70) received the vaccine outside the indicated age specified in the label (off-label). The demographic data for children who received the vaccine as per label and the off-label vaccine are mentioned in Table 1.

**Table 1.** Demographic data.

Characteristics	Vaccine as per label (N = 3040)	Vaccine off-label (N = 71)
Age (in weeks)		
Mean	9.4	16.0
SD	±1.7	±7.8
Gender n (%)		
Female	1494	31
Male	1546	40
Origin n (%)		
Korean	3036 (99.9%)	71 (100%)
Chinese	2 (0.1%)	—
Japanese	2 (0.1%)	—
Concomitant vaccination n (%)		
yes	2970 (97.7%)	70 (98.6%)
Previous medical history		
All	995 (32.7%)	29 (40.8%)
Infections and infestations	484 (15.9%)	20 (28.2%)
Skin and Subcutaneous tissue disorders	235 (7.7%)	5 (7.0%)
Pregnancy and perinatal conditions	204 (6.7%)	4 (5.6%)

SD: standard deviation.

### Safety

Among the 3040 infants, at least 1 expected AE was recorded for 926 infants (30.5%; 95% CI: 28.8–32.1%) during the 31-day follow up period after either vaccine dose, of which ≥1 ADR (any AE whose causality to the drug could not be ruled out) was reported for 261 infants (8.6%; 95% CI: 7.6−9.6%).

The most commonly seen expected AEs during the 31-day follow are shown in Table 2. Irritability was the most frequently reported expected AE, reported for 426 subjects, followed by fever, loss of appetite and vomiting. Fever was the most frequently reported expected ADR.

**Table 2.** Most common expected and unexpected adverse events and adverse drug reactions.

	Vaccine administered as per label		Vaccine administered off-label	
Symptoms	n (%; 95% CI)	Adverse Drug Reaction n (%, 95%CI)	n (%; 95% CI)	Adverse Drug Reaction n (%, 95%CI)
Most commonly seen expected AEs				
At least one symptom	926 (30.5; 28.8–32.1)	261 (8.6; 7.6–9.6)	20 (28.2; 18.1−40.1)	5 (7.0; 2.3−15.7)
Irritability	426 (14.0; 2.8−15.3)	67 (2.2; 1.7−2.7)	8 (11.3; 5.0–21.0)	–
Fever	268 (8.8; 7.8−9.9)	98 (3.2; 2.6%−3.9)	5 (7.0; 2.3−15.7)	1 (1.4; 0.0−7.6)
Loss of appetite	256 (8.4; 7.5−9.5)	27 (0.9; 0.6−1.3)	5 (7.0; 2.3−15.7)	1 (1.4; 0.0−7.6)
Vomiting	241 (7.9; 7.0−8.9)	71 (2.3; 1.8−2.9)	7 (9.9; 4.1−19.3)	3 (4.2; 0.9−11.9)
Diarrhea	119 (3.9; 3.3−4.7)	49 (1.6; 1.2−2.1)	2 (2.8; 0.3−9.8)	–
Common cold	124 (4.1%; 3.4−4.8)	6 (0.2%;0.1−0.4)	5 (7.0; 2.3−15.7)	–
Dermatitis Atopic	63 (2.1; 1.6−2.6)	2 (0.1; 0.0−0.2)	1 (1.4; 0.0−7.6)	1 (1.4;0.0−7.6)
Most commonly seen unexpected AEs				
At least one symptom	987 (32.5; 30.8−34.2)	110 (3.6; 3.0−4.3)	25 (35.2; 24.2−47.5)	3 (4.2; 0.9−11.9)
Upper respiratory tract infection	264 (8.7; 7.7−9.7)	22 (0.7; 0.5−1.1)	5 (7.0; 2.3−15.7)	–
Coughing	248 (8.2; 7.2−9.2)	20 (0.7; 0.4−1.0)	4 (5.6; 1.6−13.8)	–
Bronchiolitis	152 (5.0; 4.3−5.8)	4 (0.1; 0−0.3)	7 (9.9; 4.1−19.3)	–
Gastroenteritis	105 (3.5; 2.8−4.2)	19 (0.6; 0.4−1.0)	4 (5.6;1.6−13.8)	–
Bronchitis	89 (2.9; 2.4−3.6)	7 (0.2; 0.1–0.5)	3 (4.2; 0.9−11.9)	1 (1.4; 0.0−7.6)
Otitis media	59 (1.9; 1.5−2.5)	9 (0.3; 0.1−0.6)	1 (1.4; 0.0−7.6)	–
Rash	49 (1.6%; 1.2−2.1)	9 (0.3%; 0.1− 0.6)	2 (2.8%; 0.3−9.8)	1 (1.4%; 0.0−7.6)
Sinusitis	12 (0.4%; 0.2−0.7)	2 (0.1%; 0.0−0.2)	2 (2.8%; 0.3−9.8)	2 (2.8%; 0.3−9.8)

N = Total number of infants; n (%): number (percentage) of infants with an adverse event; 95% CI: 95% Confidence Interval.

At least 1 unexpected AE was reported for 987 infants (32.5%; 95% CI: 30.8−34.2%) during the 31-day follow-up period after either dose of RIX4414, of which ≥1 ADR was reported for 110 subjects (3.6%; 95% CI: 3.0−4.3%).

The most commonly seen unexpected AEs during the 31-day follow are shown in Table 2. Upper respiratory tract infection was the most frequent AE during the 31-day follow up as well as the most commonly reported ADR under unexpected AEs (Table 2). No case of the upper respiratory tract infection was assessed to be definitely or possibly related to vaccination. The safety data for the children who were administered the off-label vaccine have also been in presented in Table 2.

Thirty four SAEs were recorded in 24 subjects (0.8%; 95% CI: 0.5−1.2%), of which all were considered unrelated to the vaccine (Table 3). The most common SAE was bronchiolitis in 9 subjects (0.3%; 95% CI: 0.1−0.6%), followed by otitis media, urinary tract infections, gastroenteritis and pneumonia seen in 3 subjects each (0.1%; 95% CI: 0.0−0.3%). There were no deaths during this surveillance period.

**Table 3.** Serious adverse events reported through surveillance period (N = 3040).

Symptoms	n (%; 95% CI)
Fever	1 (0.0; 0−0.2)
Colitis	1 (0.0; 0−0.2)
Gastroenteritis	3 (0.1; 0−0.3)
Viral gastroenteritis	1 (0.0; 0−0.2)
Paralytic ileus	1 (0.0; 0−0.2)
Otitis media	3 (0.1; 0−0.3)
Sepsis	1 (0.0; 0−0.2)
Bronchiolitis	9 (0.3; 0.1−0.6)
Bronchitis	2 (0.1; 0−0.2)
Bronchopneumonia	1 (0.0; 0−0.2)
Croup	1 (0.0; 0−0.2)
Pneumonia	3 (0.1; 0−0.3)
Viral pneumonia	1 (0.0; 0−0.2)
Upper respiratory tract infection	1 (0.0; 0−0.2)
Cytomegalovirus gastrointestinal infection	1 (0.0; 0−0.2)
Hydronephrosis	1 (0.0; 0−0.2)
Urinary tract infection	3 (0.1; 0−0.3)

N = Total number of infants; n (%): number (percentage) of infants with an SAE; 95% CI: 95% confidence interval.

Two children administered the off-label vaccine displayed SAEs, one child was diagnosed with otitis media which was resolved in 25 days and the other child was diagnosed with pneumonia which resolved in 19 d. Both SAEs were unrelated to the vaccine.

## Discussion

This post-marketing surveillance (PMS) study assessed the safety of the RIX4414 vaccine in 3040 Korean infants in a real-life setting, as required by the MFDS regulations. During the study, which extended from September 2008 until August 2013, the original lyophilized vaccine formulation of RIX4414 was replaced by a liquid formulation. Our results indicate that both formulations of RIX4414 had no prominent safety concerns and were well-tolerated in Korean infants. Further, no vaccine-related SAEs were reported throughout the study period. Safety and tolerability of 2 doses of RIX4414 has been previously demonstrated in clinical trials conducted in various countries including Korea.^11-15,18-22^ An integrated safety summary of RIX4414 based on 8 double-blind, placebo-controlled, randomized trials also indicated safety profiles similar to those seen in our study.^20^

According to the data included in the Korean label and from pooled analysis of 17 placebo controlled trials conducted all over the World, irritability and diarrhea were commonly seen in 1 per ≥100 participants and abdominal pain, flatulence and dermatitis were less commonly seen in about 1 per ≥1000 participants.^22^ Buyse et al also collated data from 28 randomized placebo controlled trials and concluded that while a similar safety profile was observed for both the placebo and the vaccine group, irritability and coughing were the most commonly seen symptoms.^21^ In comparison, we observed in the current study that fever (98 in 3040 participants) and vomiting (71 in 3040 participants) were more commonly seen symptoms, while diarrhea (49 in 3040 participants) was less common.

The strengths of our study include the large sample size of 3040 individuals as well as the real-world setting in which it was conducted. On the other hand, the sample size may be insufficient to detect rare AEs, particularly intussusception, which has been shown to be associated with RV vaccination. These factors will contribute to a more robust result and will add to the existing safety database for RIX4414. The main limitation of this study is the lack of a control group, which may necessitate a more cautious interpretation of the results. Furthermore, while there was no pre-defined stratified analysis performed by vaccine presentation, both presentations contain the same antigen content and demonstrate similar immunogenicity. Till date, there has been no change in the safety profile of both presentations of *Rotarix*™.^23^

Our safety results from this study are in line with the observation from other studies and support the use of both formulations of RIX4414 in Korea.

## Conclusions

In conclusion, this 6 y surveillance study to monitor safety profiles of both formulations of RIX4414 shows an acceptable safety profile of the vaccine when administered to Korean infants according to the local prescribing recommendations and current clinical practice.

## Materials and methods

### Study design and population

**Figure 1.**
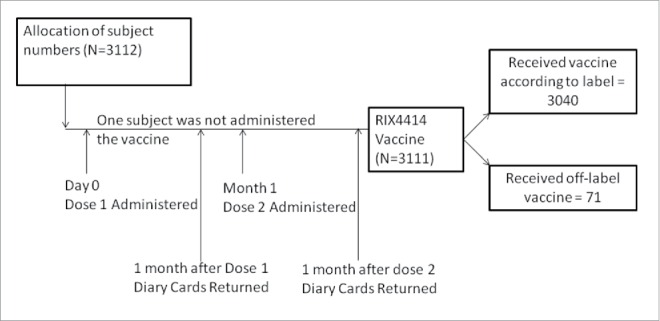
Study design.

This open, non-comparative, multicenter Post Marketing Surveillance (PMS) study was conducted at 83 centers in Korea for a period of 6 y from September 2008 to August 2013 (NCT00750893), following the approval of RIX4414 (Fig. 1).

Healthy infants aged at least 6 weeks were enrolled and received 2 doses of RIX4414. Infants who had previously received a first dose of RIX4414 could also be included, and received their second RIX4414 dose as part of the study. Infants were excluded from the study if they had any contraindications or risks described in the prescribing information for the Korean label.

### Ethics statement

The study was conducted according to the regulations of MFDS and approved by the independent ethics committee at each study center. Parents/guardians provided written informed consent before study enrollment.

### Vaccination

Each dose of either formulation of RIX4414 vaccine contained ≥10^6.0^ CCID_50_ (median Cell Culture Infective Dose). Two doses of RIX4414 (lyophilized or liquid formulations) were administered orally according to standard clinical practice and regulations.^24^ Following vaccination, infants were observed for at least 30 minutes in case of rare anaphylactic reactions. The first dose was given to infants from 6 weeks of age and the second dose was given at least 4 weeks later (before 24 weeks of age).

## Assessments

Baseline demographic data were collected by the investigators at the first visit.

Parents/guardians were given diary cards to record adverse events (AEs) for 31 d following each dose of vaccination. For the first 2 y of the study, AEs were recorded as solicited (8-day follow-up) and unsolicited (31-day follow-up), but due to changes made to the *Guidelines for the Korean New Drug Re-examination* by the MFDS, all AEs were recorded for 31 d after each vaccine dose from Years 3−6. All AEs were categorized as ‘Expected’ or ‘Unexpected’ according to the approved Korean Prescribing information.^22^ AEs were further categorized based on causality, and an adverse drug reaction (ADR) was defined as ‘any AE whose causality to the drug could not be ruled out’. AEs were classified as definitely/certain, probable/likely, possible, unlikely, conditional/unclassified and unassessible/unclassifiable.

The intensity of AEs were classified as: mild (easily tolerated by the infant, causing minimal discomfort and not interfering with everyday activities), moderate (sufficiently discomforting to interfere with normal everyday activities) and severe (prevents normal, everyday activities).

Serious Adverse Events (SAEs) were recorded for the entire surveillance period, which was approximately 3 months for each infant.

The investigator was responsible for the classification of AEs and SAEs as defined in the protocol as well as assessing the causality of the AEs based on the requirements by the MFDS in Korea.

### Statistical analysis

Statistical analysis, using Statistical Analysis System (*SAS*) version 9.2, was performed on the Total Vaccinated Cohort (TVC), which comprised children who received at least 1 dose of the vaccine during the surveillance period. AEs and SAEs were analyzed according to whether they were expected or unexpected, using criteria in the WHO adverse reactions terminology.^25^ The overall incidence (for all causality grades, as mandated by the MFDS), with exact 95% confidence intervals (CI), of any AEs occurring within 31 d was tabulated for both according to label and off-label use of the vaccine. SAEs and withdrawals due to AEs reported during the PMS period were also described.

## Trademark statement

*Rotarix* is a trademark of the GSK group of companies. *Rotateq* is a trademark of the Merck & Co. USA.

## Abbreviations

RV: Rotavirus
GE: Gastroenteritis
PMS: Post-Marketing Surveillance
CCID: Cell Culture Infective Dose
AE: Adverse Events
MFDS: Ministry of Food and Drug Safety
ADR: Adverse Drug Reaction
SAE: Serious Adverse Event
SAS: Statistical Analysis Software
TVC: Total Vaccinated Cohort
CI: Confidence Interval
